# Identification of disulfidptosis related subtypes, characterization of tumor microenvironment infiltration, and development of DRG prognostic prediction model in RCC, in which *MSH3* is a key gene during disulfidptosis

**DOI:** 10.3389/fimmu.2023.1205250

**Published:** 2023-06-23

**Authors:** Kai Xu, Ye Zhang, Zhiwei Yan, Yuchan Wang, Yanze Li, Qiangmin Qiu, Yang Du, Zhiyuan Chen, Xiuheng Liu

**Affiliations:** ^1^ Department of Urology, Renmin Hospital, Wuhan University, Wuhan, Hubei, China; ^2^ Institute of Urologic Disease, Renmin Hospital, Wuhan University, Wuhan, Hubei, China; ^3^ School of Science, Hubei University of Technology, Wuhan, China

**Keywords:** RCC, disulfidptosis, tumor microenvironment, prognosis, immunotherapy

## Abstract

Disulfidptosis is a newly discovered mode of cell death induced by disulfide stress. However, the prognostic value of disulfidptosis-related genes (DRGs) in renal cell carcinoma (RCC) remains to be further elucidated. In this study, consistent cluster analysis was used to classify 571 RCC samples into three DRG-related subtypes based on changes in DRGs expression. Through univariate regression analysis and LASSO-Cox regression analysis of differentially expressed genes (DEGs) among three subtypes, we constructed and validated a DRG risk score to predict the prognosis of patients with RCC, while also identifying three gene subtypes. Analysis of DRG risk score, clinical characteristics, tumor microenvironment (TME), somatic cell mutations, and immunotherapy sensitivity revealed significant correlations between them. A series of studies have shown that *MSH3* can be a potential biomarker of RCC, and its low expression is associated with poor prognosis in patients with RCC. Last but not least, overexpression of *MSH3* promotes cell death in two RCC cell lines under glucose starvation conditions, indicating that *MSH3* is a key gene in the process of cell disulfidptosis. In summary, we identify potential mechanism of RCC progression through DRGs -related tumor microenvironment remodeling. In addition, this study has successfully established a new disulfidptosis-related genes prediction model and discovered a key gene *MSH3*. They may be new prognostic biomarkers for RCC patients, provide new insights for the treatment of RCC patients, and may inspire new methods for the diagnosis and treatment of RCC patients.

## Introduction

Renal cell carcinoma (RCC) is the most common subtype of renal cancer worldwide, ranking second among the most common malignant tumors in the urogenital system. The characteristics of RCC are asymptomatic, high mortality, high recurrence rate, easy to metastasize, and easy to develop treatment tolerance. This makes many patients with RCC already advanced at the time of diagnosis, and the prognosis is often poor (1–3). Fortunately, with the rapid development of medical standards and medical research worldwide, the efficacy of treating RCC has significantly improved. However, the overall 5-year survival rate (OS) of RCC patients is still not ideal. Worse still, even patients with RCC who have undergone surgical treatment may experience metastasis and recurrence after surgery. Some literature indicates that the 5-year OS of patients with metastatic RCC is even less than 10% (4–8). Therefore, it is urgent to find new prognostic biomarkers that can effectively predict the prognosis of patients with RCC and provide new therapeutic insights for RCC treatment.

Regulated cell death (RCD) is a type of cell death that can be regulated by controlling specific molecular pathways or conducting genetic and pharmacological processing (9). Identification and phenotyping of cell death mechanisms can not only promote a basic understanding of cell homeostasis but also provide important ideas for the treatment of various diseases such as cancers. Disulfidptosis is a newly discovered mode of cell death induced by disulfide stress, mainly due to the depletion of intracellular reduced nicotinamide adenine dinucleotide phosphate (*NADPH*), resulting in the accumulation of cystine, followed by the initiation of actin cytoskeletal disulfide bonding and cytoskeletal contraction, ultimately inducing disulfide toxicity, as revealed by the research results recently published by Liu et al. (10).

As early as six years ago, researchers found that *SLC7A11* significantly promotes cell death under glucose starvation. Contrarily, the uptake of cystine mediated by member 11 of the solute carrier family (*SLC7A11*; also known as *xCT*) is crucial in promoting glutathione biosynthesis, inhibiting oxidative stress, and inhibiting the occurrence of ferroptosis (11–13). In response, Liu et al. published a study (14) that found that the *SLC7A11*-mediated process of uptake of cystine and reduction to cysteine is highly dependent on the reduced nicotinamide adenine dinucleotide phosphate produced by the glucose pentose phosphate pathway. Therefore, under glucose starvation conditions, *NADPH* is greatly consumed in the *SLC7A11* high expression cells, and disulfides such as cystine are abnormally accumulated, leading to disulfide stress and rapid cell death. However, it was not until a few months ago that the mechanism of how disulfide stress triggers cell death was clarified. By collecting relevant literature to obtain some DRGs, we believe that this may have a new perspective on the prognosis and treatment of RCC patients.

Due to the novelty of the defective disulfidptosis, the relevant research is not comprehensive. However, our study firstly explore the role of disabling disulfidptosis in RCC and its relationship with tumor microenvironment infiltration and has established a risk prediction model. In our study, we downloaded mRNA expression profiles, clinical data, and somatic variation data from the Cancer Genome Atlas (TCGA) and Gene Expression Omnibus (GEO) databases for RCC patients. First, we extracted the expression amount of DRGs, identified three DRG-related subtypes through consistency cluster analysis, and screened the differentially expressed genes (DEGs) among the subtypes. Based on these DEGs, we genotyped again to obtain three genotypes. Based on these differentially expressed genes, we then established a prognostic prediction model that can effectively predict the prognosis of patients with RCC. At the same time, the tumor microenvironment infiltration, immunotherapy sensitivity, and somatic cell variability of samples with different subtypes and different risks were characterized. Through a series of analyses and validation, the prediction effect of the model is evaluated. Finally, during the analysis of the model gene, we also found an important key gene *MSH3* and conducted relevant analysis and experimental verification to investigate the possibility of its promotion of disulfidptosis in RCC cell lines. In summary, we believe that disabling disulfidptosis may be a potential biological target for the diagnosis and treatment of RCC, and our research results can provide a new perspective for the diagnosis and treatment of RCC.

## Materials and methods

### Data collection

A total of 580 patients with RCC from two independent data sets (TCGA-KIRC, GSE29609) were included in this study. The mRNA expression profile, clinical data, and somatic cell variation data of 541 RCC patients and 72 normal human renal tissues were downloaded from the TCGA database (https://portal.gdc.cancer.gov/). The clinical data included age, gender, histological grade, pathological stage, pathological T stage, pathological M stage, pathological N stage, survival time, and survival status. In addition, the gene expression data files and corresponding clinical information files of 39 RCC patients were downloaded from the GEO database (https://www.ncbi.nlm.nih.gov/geo/). Collate these raw data through the limma program (15) package of R software, normalize the gene expression file, and standardize it to fragments with a million expression levels per thousand bases. 24 DRGs were obtained from previous studies (10–14) and their expression levels were extracted from the collated gene expression files, the specific genes are shown in **Table S1**. It should be stated that our study excluded nine RCC samples with incomplete clinical data. The above data were downloaded through the official website, in full compliance with the access policies for TCGA and GEO databases, and strict compliance with the publication guidelines.

### Consistency clustering analysis based on DRGs expression

Based on the expression level of DRGs in each sample, we were able to classify 571 RCC samples into discrete molecular clusters by using the ConsensusClusterPlus (16) package of R software. Kaplan-Meier survival analysis was used to investigate the clinical utility of DRGs in RCC patients, and survival curves was plotted by the survival and survminer package of R software. Meanwhile, principal component analysis (PCA) was performed by the ggplot2 (17) program package of R software. The ESTIMATE (18) and CIBERSORT (19) algorithms were used to calculate the percentage of immune and stromal cells in RCC patients, and the enrichment fraction of each immune cell infiltration in RCC patients was assessed by the single-sample gene set enrichment analysis (ssGSEA) algorithm (20).

### Survival analysis between different subtypes and functional enrichment analysis of DEGs

The Kaplan-Meier method was used to study the differences in OS among different subtypes of RCC patients. Through the limma package of R software, we screened the DEGs among subtypes. To further understand the functions and pathways involved in DEGs, we also conducted functional enrichment analysis, including Gene Ontology (GO) enrichment analysis and Kyoto Encyclopedia of Genes and Genomes pathway (KEGG) enrichment analysis.

### Gradual construction and verification of DRG prediction model

We randomly divided 571 RCC samples into two groups in a one-to-one ratio, one as a training set (n=285) and the other as a verification set (n=286). Firstly, univariate regression analysis was conducted based on the OS of the training set samples, thereby screening out DEGs with significant prognostic value. Next, through LASSO-Cox regression analysis and the glmnet (21) package of R software, the possibility of overfitting is minimized and genes with potentially high correlation with other genes are excluded. Finally, a prognostic prediction model was established through multivariate Cox regression analysis. The risk score is calculated based on the expression level and regression coefficient of the genes in the results, and the calculation formula is as follows:


Risk score=∑(expression of gene*coef)

The calculated risk score is taken as the median value, and each group is divided into a high-risk group and a low-risk group. The Kaplan-Meier analysis was used to assess survival differences between the high-risk and low-risk groups. Use the timeROC package of R software to draw 1-year, 3-year, and 5-year receiver operating characteristic (ROC) curves, and calculate the corresponding time-dependent area under the curve (AUC) to evaluate the accuracy of model predictions. Subsequently, the correlation between clinical data and risk scores of RCC patients was visualized in the TCGA-KIRC dataset, and the progression-free survival (PFI) of each patient was estimated based on the pan-cancer file in the TCGA database.

### Construction and evaluation of the nomogram

A nomogram was created using the rms package of R software, listing risk scores and other prognostic indicators as prognostic factors. The scores for each prognostic factor were summed, and the 1-year, 3-year, and 5-year survival probabilities of patients were predicted based on the overall score. In addition, we have also plotted calibration curves, ROC curves, and decision curve analysis (DCA) curves. In addition, we also treat risk score as an independent variable and conduct univariate and multivariate independent prognostic analysis together with other clinical traits with prognostic significance to demonstrate that risk score can be used as an independent prognostic factor to independently predict the prognosis of patients with RCC.

### Analysis of tumor microenvironment, immune cell infiltration, and immune function

The CIBERSORT algorithm is used to calculate the infiltration of 23 types of immune cells in the tumor microenvironment of each sample, compare the different subsets of immune cells between the high-risk and low-risk groups, and map the infiltration patterns of immune cells related to risk scores. Subsequently, we also conducted immune function scoring, showing differences in the scoring of different immune functions between high-risk and low-risk groups. Through TIDE scoring and TME scoring, explore whether there are differences in immunotherapy sensitivity and tumor microenvironment infiltration in samples under different groups.

### Joint analysis of risk groups, typing results, somatic variation, functional enrichment, and drug sensitivity

We combined the previous classification results and risk scores of 571 RCC patients and visualized the difference in scores between different classifications using Sangi charts and box charts to verify the accuracy of the previous survival analysis results between different classifications. We used the maftools (22) program package of R software to analyze somatic mutations in RCC patients, visualizing and comparing genes with somatic mutations in high-risk and low-risk groups using waterfall diagrams. Through Gene Set Enrichment Analysis (GSEA) and Gene Set Variation Analysis (GSVA) (23), we have demonstrated the rich functions of high-risk and low-risk groups, respectively. Subsequently, we conducted gene differential expression analysis between the two groups and visualized the genes with differences through GO and KEGG enrichment analysis. Using the oncoPredict (24) algorithm of R software, we calculated the half-maximal inhibition concentrations (IC50) of commonly used drugs in different groups of RCC patients as an indicator of drug sensitivity.

### Comprehensive bioinformatics analysis of the key gene *MSH3*

We first demonstrated the expression of five model genes in RCC patients, then plotted their molecular correlation loops, and described the changes in the remaining four model genes as the expression of *MSH3* changes. Using a box graph to visualize the expression level of *MSH3* in RCC patients, and using Kaplan Meier survival analysis to demonstrate the OS of RCC patients under both *MSH3* expression modes, we also plotted a 5-year ROC curve to assess the accuracy of *MSH3* in predicting the prognosis of RCC patients. In addition, we also combined *MSH3* expression levels with clinical data from RCC patients to visualize statistically significant clinical traits using a box graph. In addition, we also conducted correlation analysis of immune checkpoints, immune cell infiltration analysis, immunotherapy sensitivity analysis, correlation analysis of *NCKAP1* expression level, GO enrichment analysis, KEGG enrichment analysis, and GSEA enrichment analysis related to *MSH3* expression level.

### Cell culture

Both human proximal tubular epithelial cells (HK-2) and human renal cell carcinoma cell lines (786-O and A498) are purchased from ATCC. The HK-2 cell line was cultured in DMEM/F-12 medium (cytiva) supplemented with 10% fetal bovine serum (gibco) and 1% penicillin/streptomycin (biosharp). The 786-O and A498 cell lines were cultured in RPMI 1640 medium (cytiva) supplemented with 10% fetal bovine serum and 1% penicillin/streptomycin. All cell lines were cultured in an incubator with a constant temperature of 37 °C and 5% CO2.

### Transfection

The pcDNA3.1 plasmid for *MSH3* and *SLC7A11* overexpression was purchased from Sangon Biotech (Shanghai). RCC cells were seeded in 6-well plates at an appropriate density. After 12 hours, transfection was performed using lipofectamine 3000 (L300001, Thermo Fisher Scientific, USA) according to the manufacturer’s instructions.

### Cell death assay

Cells were inoculated in 12-well plates the day before the treatment according to the manufacturer’s instructions. After incubation with special media with or without appropriate drugs, cells were trypsin-digested and collected in 1.7 ml microtubes, washed once with PBS and resuspended in PI in 1ug/ml cold PBS. Finally, we assessed cell death by flow cytometry (BD Biosciences).

### ATP level detection

According to the manufacturer’s instructions, we use an ATP assay colorimetric kit (ab83355, Abcam, UK) to measure ATP levels. The ATP assay kit is based on the phosphorylation of glycerol, producing a product that can be subjected to colorimetric quantification (OD=570nm).

### Western blot assays

First, we homogenized HK-2 cells and RCC cells in RIPA lysis buffer containing protease inhibitors. Then, we measured the protein concentration of each sample using bicinchoninic acid (BCA) and then separated it using SDS-PAGE. The protein of each sample was transferred to a polyvinylidene fluoride transfer membrane and sealed with 5% skimmed milk for 1 hour. The membrane was incubated with antibodies to resist *MSH3* (ab69619, Abcam, UK), *SLC7A11* (ab175186, Abcam, UK) and glyceraldehyde 3-phosphate dehydrogenase (*GAPDH*; ab8245, Abcam, UK). After incubating with the first antibody overnight, the membrane was washed and incubated with the second antibody. Protein bands were visualized using enhanced chemiluminescence reagents (WP20005, Thermo Fisher Scientific, USA).

### Statistical analysis

All data in this study are expressed as mean standard deviation (SD). All bioinformatics analysis is performed using R software (v.4.2.2). Statistical analysis was conducted using GraphPad Prism analysis software. P<0.05 is considered to have a statistically significant difference, and * indicates p<0.05, ** represents p<0.01, and *** indicates p<0.001.

## Results

### Differential expression and prognostic value of 24 DRGs in RCC

**Figure 1:**
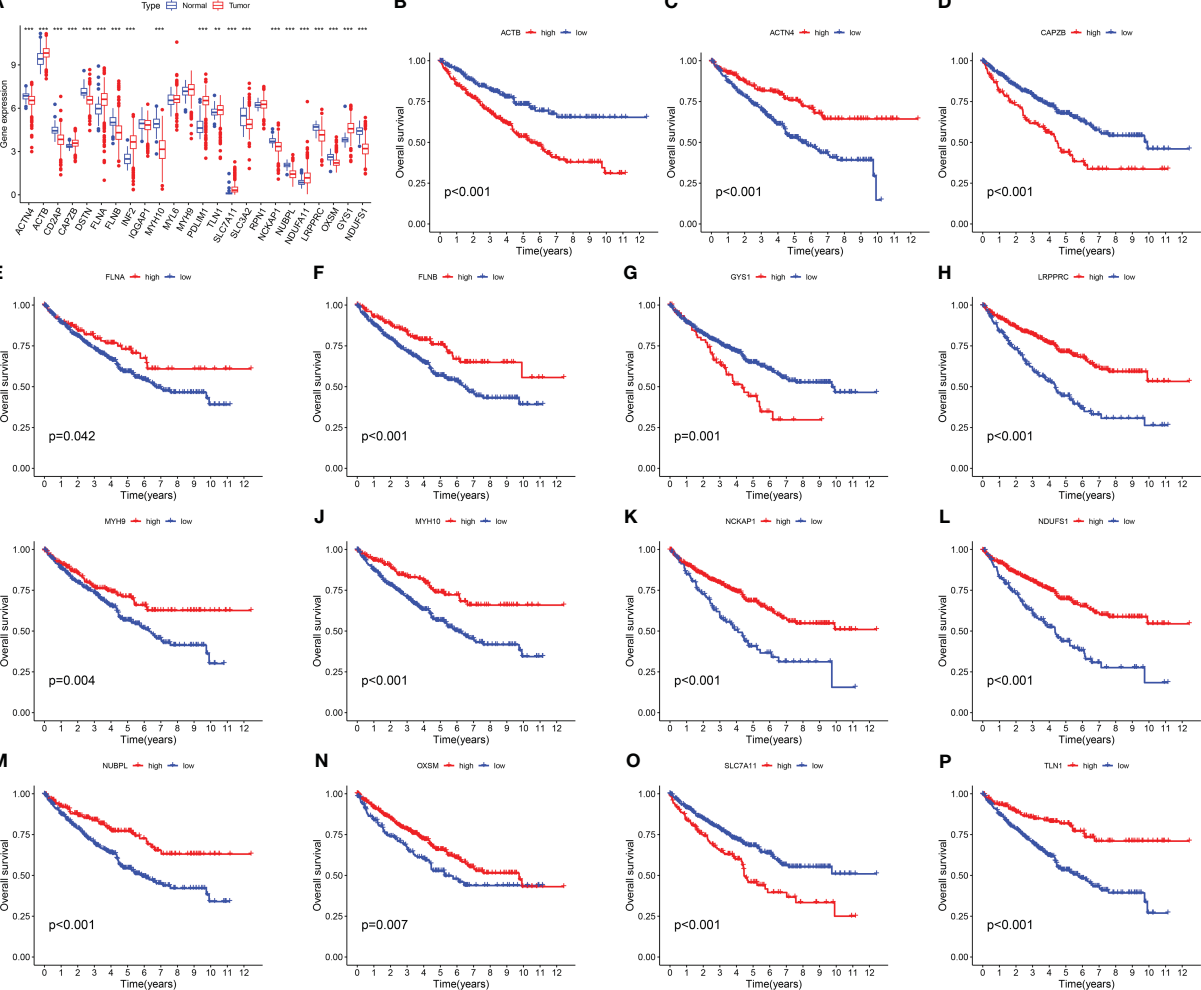
Expression levels and Kaplan-Meier survival analysis of 24 DRGs in the TCGA-KIRC dataset. **(A)** The expression level of 24 DRGs in the TCGA-KIRC dataset. **(B–P)** The association between the selected 15 DRGs and the OS of RCC patients. The symbol ** indicates p<0.01, and *** indicates p<0.001.

We investigated the expression levels of 24 DRGs in 541 RCC patients and 72 normal human kidney tissues obtained from the TCGA-KIRC dataset, and the results showed that the majority of the 24 DRGs were differentially expressed between the tumor and normal groups (**Figure 1A**). Kaplan-Meier survival analysis was performed for 24 DRGs in RCC, and we found that the expression of 21 of them was closely associated with OS in RCC patients, and we showed only 15 of them (**Figures 1B–P**; **Table S2**). *ACTN4, FLNA, FLNB, LRPPRC, MYH9, MYH10 NCKAP1, NDUFS1, NUBPL, OXSM*, and *TLN1* had a better OS in patients with high expression, and conversely, patients with high expression of *ACTB, CAPZB, GYS1*, and *SLC7A11* had a poorer OS.

### Identification of disulfidptosis subtypes and genetic subtypes in RCC

**Figure 2:**
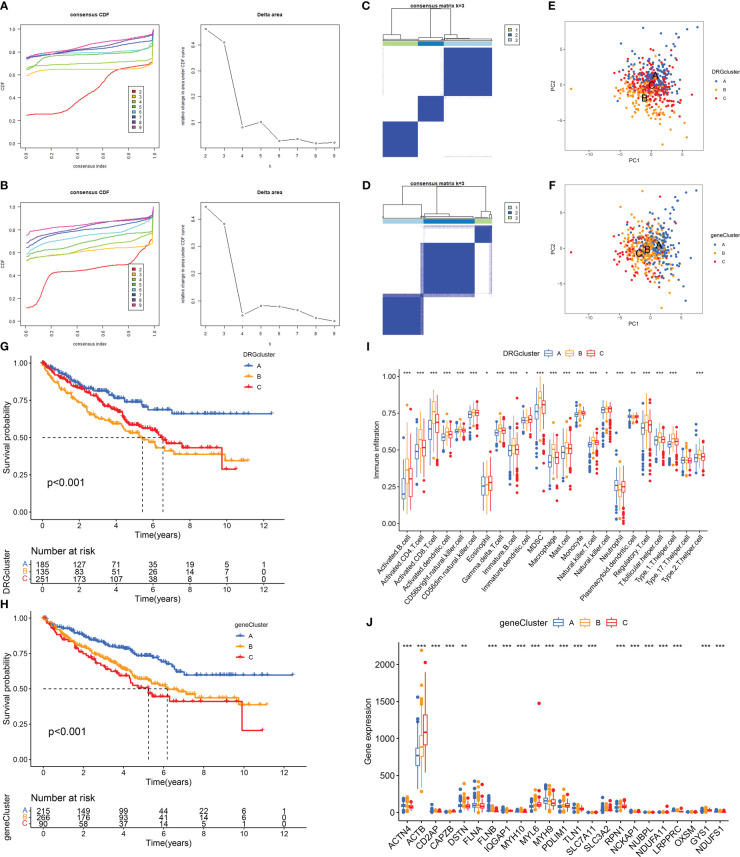
Determination and correlation analysis of RCC subtypes. **(A, B)** The CDF distribution diagram and Delta area diagram of consistency clustering analysis shows the optimal κ Value. **(C, D)** The consistency matrix diagram shows that when κ Consistency clustering between samples when taking the optimal value. **(E, F)** The images of PCA show that the unsupervised consistency clustering method is effective in classifying RCC. **(G, H)** The results of the Kaplan-Meier survival analysis showed that the survival rate of patients with RCC varied among different subtypes. **(I)** Results of immune cell infiltration between different subtypes in the DRGCluster. **(J)** The expression level of DRGs among different subtypes in the geneCluster. The symbol * indicates p<0.05, ** indicates p<0.01, and *** indicates p<0.001.

**Figure 3:**
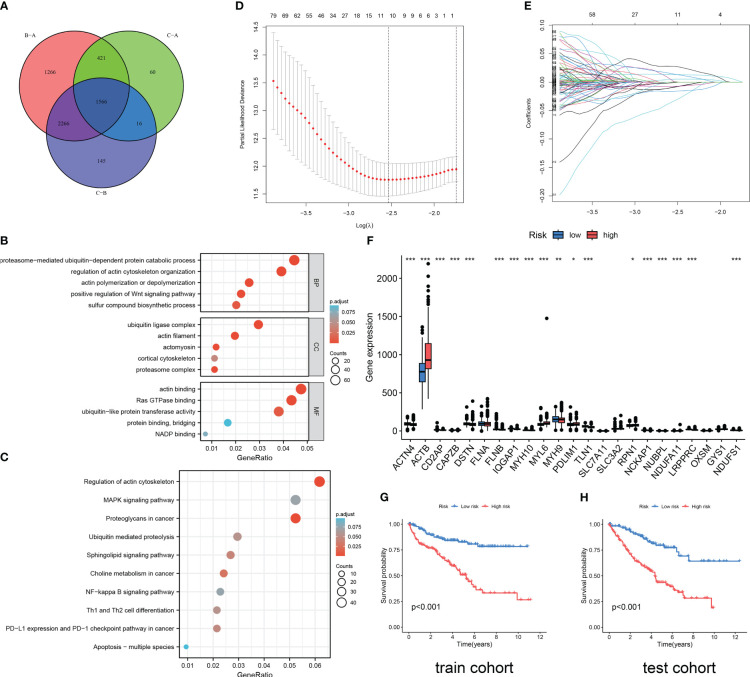
Functional enrichment analysis and DRG prediction model construction based on DEGs. **(A)** The intersection of DEGs between three DRG types. Results of GO enrichment analysis **(B)** and KEGG enrichment analysis **(C)** for 1566 DEGs. **(D, E)** The coefficient distribution of LASSO-Cox regression analysis and adjustment parameters were calculated based on partial likelihood deviation and ten-fold cross-validation. **(F)** The expression level of DRGs in RCC patients in different risk groups. The model predicts the survival rates of the training cohort **(G)** and the validation cohort **(H)** through Kaplan Meier survival analysis. The symbol * indicates p<0.05, ** indicates p<0.01, and *** indicates p<0.001.

Disulfidptosis typing of 571 RCC samples from the TCGA-KIRC and GSE29609 datasets according to the expression levels of DRGs, the consensus cumulative distribution function (consensus CDF) plot shows the CDF distribution under a different number of clusters κ. The CDF distribution when κ=3 is flatter and approximates to the maximum, while the area under the CDF curve at κ=4 is relatively less obvious than that at κ=3. Therefore, we choose the clustering result at κ=3 (**Figure 2A**) and divide the samples into three disulfidptosis subtypes (**Figure 2C**). The PCA results showed that the three subtypes were well differentiated (**Figure 2E**). The 1566 DEGs between the three subtypes were identified by limma package (**Figure 3A**; **Table S3**), and similarly, the 571 RCCs were then genotyped according to the expression level of DEGs, and the results of CDF distribution and Delta area plots indicated the optimal clustering results at κ=3 (**Figure 2B**). The consistency matrix (**Figure 2D**) and PCA results (**Figure 2F**) of the three genetic subtypes showed good discrimination. The results of survival analysis showed that the longest OS among disulfidptosis subtypes was found for subtype A (n=185), followed by subtype C (n=251), and the worst was found for subtype B (n=135) (**Figure 2G**). While the worst OS among genotypes was subtype C (n=90), followed by subtype B (n=266), and the best was subtype A (n=215) (**Figure 2H**). According to the results of the CIBERSORT method, there was a significant difference in immune cell infiltration between the three disulfidptosis subtypes (**Figure 2I**). Activated B cells, activated CD4 T cells, activated CD8 T cells, activated dendritic cell, CD56bright natural killer cell, CD56dim natural killer cell, Gamma delta T cell, Immature B cell, MDSC, Macrophage, Mast cell, Monocyte, Natural killer T cell, Regulatory T cell, T follicular helper cell, Type 1 T helper cell, Type 2 T helper cell had the highest level of infiltration in fraction A, Eosinophil, Immature dendritic cell, and Natural killer cell was the highest level of infiltration in fractal C, while Neutrophil and Plasmacytoid dendritic cell were the highest levels of infiltration in fractal B. In addition, we also performed a differential analysis of the expression levels of DRGs between the three genotypes, and the results showed that 21 DRGs showed great expression differences between the three genetic subtypes (**Figure 2J**).

### Construction of a predictive risk model based on DEGs associated with subtypes

As shown in **Figure 3A**, 1566 DEGs were obtained by crossing the differential gene sets of three subtypes. Then, we conducted GO (**Figure 3B**) and KEGG (**Figure 3C**) analysis on these 1566 DEGs. Proteasome-mediated ubiquitin-dependent protein catabolic process, regulation of actin cytoskeleton organization, actin polymerization or depolymerization, positive regulation of WNT signaling pathway, and sulfur compound biosynthetic process is the main enriched in BP terms. Ubiquitin ligase complex, actin filament, actomyosin, cortical cytoskeleton, and proteasome complex are the main enriched in CC terms. Actin binding, Ras GTPase binding, ubiquitin-like protein transferase activity, protein binding, bridging, and NADP binding are the main enriched in MF terms. Regulation of actin cytoskeleton, MAPK signaling pathway, Proteoglycans in cancer, Ubiquitin mediated proteolysis, Sphingolipid signaling pathway, Choline metabolism in cancer, NF-kappa B signaling pathway, Th1 and Th2 cell differentiation, *PD-L1* expression and *PD-1* checkpoint pathway in cancer, Apoptosis-multiple species are the main enriched in KEGG pathways. Then, through the Caret package of R software, 571 samples were randomly divided into train cohort (n=285) and test cohort (n=286) in a one-to-one ratio. Through univariate Cox regression analysis of 1566 DEGs, 1187 OS-related genes were screened. Subsequently, we conducted LASSO Cox regression analysis on 1187 OS-related genes (**Figures 3D, E**), and finally identified five genes to construct a DRG risk model (**Tables S6**, **S7**):


$$\begin{array}{l} \text{DRG risk score}=\left(-0.15489*MSH3\text{ expression}\right)+\left(-0.05347*CRB3\text{ expression}\right)\\ +\left(0.02199*AUP1\text{ expression}\right)+\left(0.05367*RNF10\text{ expression}\right)\\ +\left(-0.03476*ELF1\text{ expression}\right). \end{array}$$

Each cohort was divided into high-risk and low-risk groups based on the median risk score. The difference analysis of the expression levels of 24 DRGs between the two groups showed that there were differences in the expression of 19 DRGs between the high-risk and low-risk groups (**Figure 3F**). The results of the survival analysis revealed that RCC patients in the high DRG risk group had worse OS than those in the low DRG risk group, both in the training cohort and the test cohort (**Figures 3G, H**). These results suggest that the DRG risk model and model-related genes are key prognostic markers for patients with RCC.

### Correlation analysis between clinical traits, survival status, and risk score

**Figure 4:**
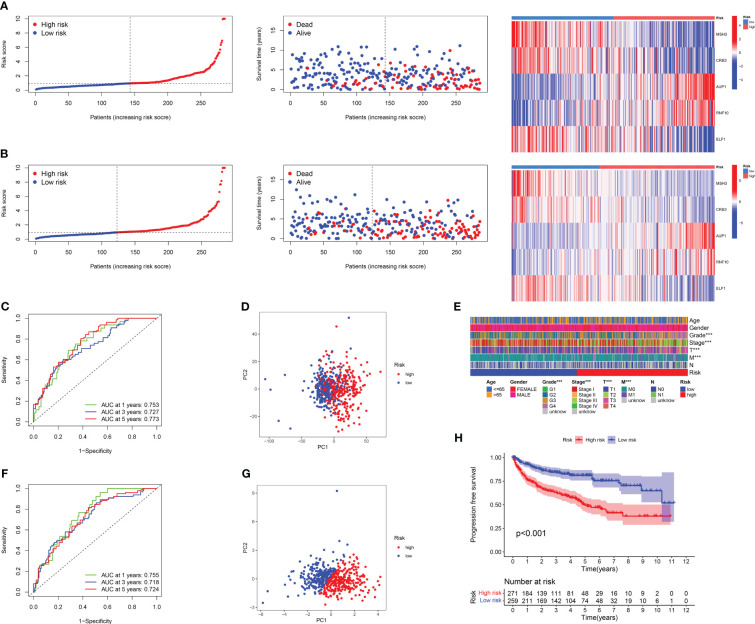
Results of correlation analysis between survival status, DRG score, and clinical characteristics. The risk curve, survival status, and model gene expression levels of RCC patients under different risk groups were displayed in the training set **(A)** and the validation set **(B)**, respectively. **(C)** The ROC curve was used to evaluate the accuracy of models in predicting prognosis in patients with RCC in a training cohort. **(D)**The PCA result of using 24 DRGs without any treatment to distinguish RCC patients. **(E)** The heat map of the correlation between important clinical features and risk score in TCGA-KIRC cohort. **(F)** The ROC curve was used to evaluate the accuracy of models in predicting prognosis in patients with RCC in the test cohort. **(G)** The PCA result of using the DRG risk model to distinguish RCC patients. **(H)** The PFI survival curves for different risk groups in TCGA-KIRC cohort.

The results of risk curves and survival status plots showed a strong positive correlation between patient survival status and risk score, and consistent differential expression of the five model genes between the high-risk and low-risk groups, both for the training cohort (**Figure 4A**; **Table S4**) and the test cohort (**Figure 4B**; **Table S5**). The ROC curves over time revealed good accuracy of the DRG risk model in predicting the 1 years, 3 years, and 5 years survival rates for the training cohort (**Figure 4C**) and test cohort (**Figure 4F**). The PCA results showed that compared to the 24 DRGs directly used to differentiate the RCC samples (**Figure 4D**), our DRG risk model was able to accurately distinguish between the high-risk and low-risk groups of RCC samples (**Figure 4G**). In addition, we investigated the relationship between risk scores and clinical traits in combination with patients’ clinical traits, and the results showed that patients with advanced RCC had higher risk scores (**Figure 4E**). The results of predicting the PFS of patients based on the pan-cancer file of the TCGA database showed that patients in the high-risk group had a worse PFS (**Figure 4H**). For the above results, we conclude that there is a significant association between DRG risk score and RCC patients in terms of histological grade, pathological stage, and prognosis.

### Development of a nomogram, evaluation of predictive effects, and independent prognostic analysis

**Figure 5:**
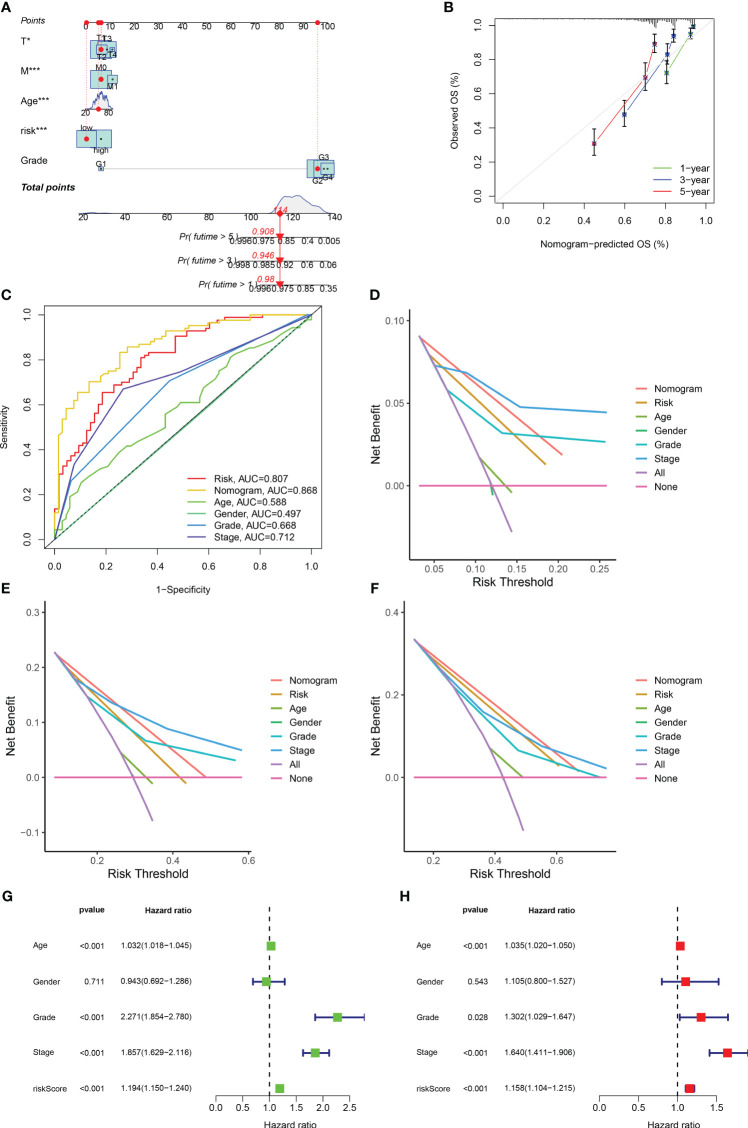
Development of a nomogram and independent prognostic analysis of risk score. **(A)** Using the prognostic clinical characteristics and risk score of RCC patients as elements in the development of a nomogram, a nomogram was drawn. **(B)** A calibration curve was used to evaluate the accuracy of the nomogram in predicting the survival probability of RCC patients. **(C)** At the same time, ROC curves are plotted for all elements in the construction nomogram, and AUC values are calculated. **(D–F)** The DCA decision curve evaluates the superiority of the nomogram in predicting the 1-year **(D)**, 3-year **(E)**, and 5-year **(F)** survival probabilities of patients with RCC by integrating all factors. **(G)** Univariate independent prognostic analysis of risk score as a prognostic factor in patients with RCC. **(H)** Multivariate independent prognostic analysis of risk score as a prognostic factor in patients with RCC. The symbol * indicates p<0.05 and *** indicates p<0.001.

Based on the obtained clinical data files and risk files, we drew a nomogram through the rms package of R software to predict the likelihood of patient survival at 1-year, 3-year, and 5-year (**Figure 5A**; **Table S8**). The results of calibration plots and ROC curves revealed that the nomogram achieved satisfactory accuracy in predicting survival (**Figures 5B, C**). The results of DCA decision curves showed that the nomogram was the most effective in predicting the likelihood of patient survival at 5 years (**Figures 5D–F**). Following this, we combined risk scores with clinical traits in univariate and multivariate Cox regression analyses, and the results showed that the five gene-based risk model, age, histological grade, and pathological stage were all independent prognostic factors (**Figures 5G, H**).

### Immune landscape and immunotherapy sensitivity based on DRG risk score

**Figure 6:**
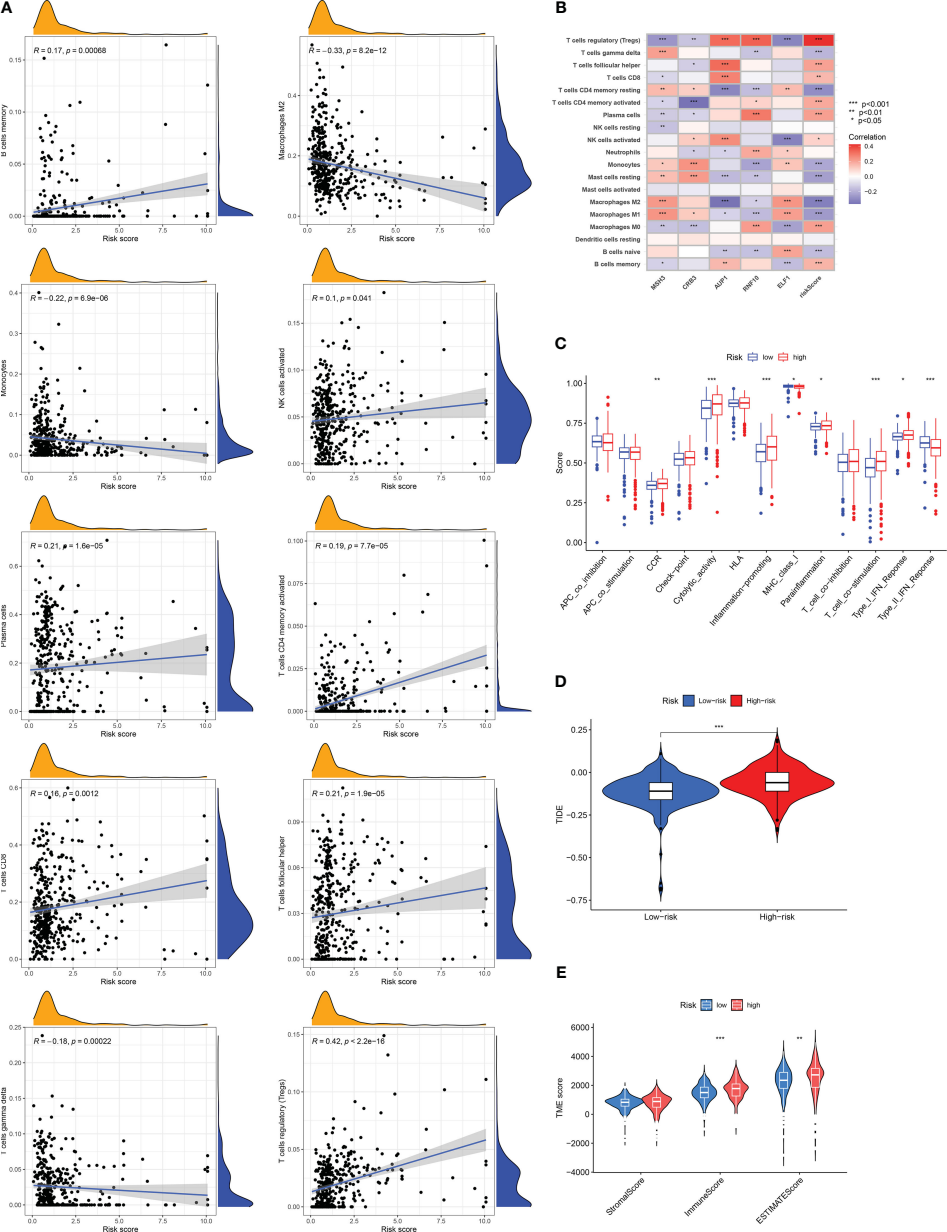
Risk score related tumor microenvironment infiltration results, immune function analysis results, and TIDE score results. **(A)** A graph showing the correlation between the content of ten selected immune cells and risk score. **(B)** Results of correlation analysis between model genes and immune cell infiltration results. **(C)** Analysis of differences in immune-related functions under different risk groups. **(D)** Differences in TIDE scores among RCC patients in different risk groups. **(E)** Results of differences in stromal cell score, immune cell score, and comprehensive score among RCC patients under different risk groups. The symbol * indicates p<0.05, ** indicates p<0.01, and *** indicates p<0.001.

According to the correlation between DRG risk score and immune cell infiltration displayed by the CIBERSROT algorithm, we only selected ten types of immune cells, and the results showed that DRG risk score was positively correlated with B cells memory, NK cells activated, Plasma cells, T cells CD4 memory activated, T cells CD8, T cells follicular helper, T cells regulation (Tregs), and negative correlations with Macrophages M2, Monocytes, T cells gamma delta (**Figure 6A**; **Table S9**). The five model genes also showed a strong correlation with immune cell infiltration (**Figure 6B**). The results of immune function enrichment analysis showed that the high-risk group was enriched on CCR, Cytolytic-activity, Inflammation-promoting, Parainflammation, T-cell-co-stimulation, and Type-I-IFN-Response, while the low-risk group was enriched on MHC-class-I and Type-II-IFN-Response (**Figure 6C**). The TIDE score assessed that the high-risk group had a higher likelihood of immune evasion, indicating that the patients had a lower likelihood of benefiting from immunotherapy (**Figure 6D**). The results of TME scoring showed that there was a significant difference between the immune cell score and the comprehensive score between the high-risk and low-risk groups, while the stromal cell score was not significant (**Figure 6E**).

### Relationship between typing results, somatic mutations, risk groups and functional enrichment analysis

**Figure 7:**
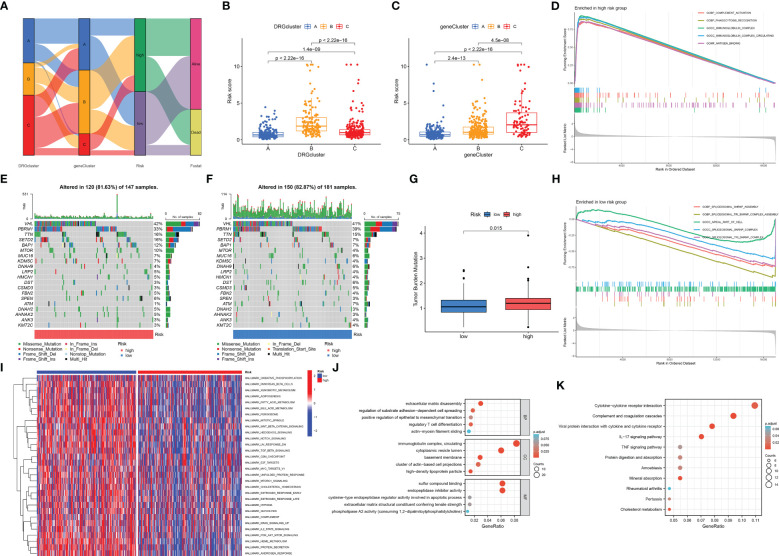
Correlation analysis of cluster results, somatic cell variation analysis, and functional enrichment analysis under different risk groups. **(A)** The Sankey diagram shows the relationship between DRGCluster, geneCluster, risk level, and survival status. **(B)** In the DRGCluster, there are differences in risk score among different clusters. **(C)** In the geneCluster, there are differences in risk score among different clusters. **(E, F)** The waterfall diagram shows the genes that most frequently undergo somatic mutations under different risk groups. **(G)** There were differences in tumor mutation load among different risk groups. **(D)** The main enriched functions of RCC patients in the high-risk group. **(H)** The main enriched function of RCC patients in the low-risk group. **(I)** The GSVA results demonstrate pathways that differ in the enrichment of RCC patients between the high-risk and low-risk groups. The DEGs between the high-risk and low-risk groups are mainly enriched in GO **(J)** and mainly enriched in KEGG **(K)**.

The Sankey diagram shows the proportion of patients in the DRG risk subgroup based on the three disability phenotypes, the three genotypes, and the DRG risk subgroup (**Figure 7A**). Different subtypes have different risk scores. In the disadvantageous classification, type B has the highest risk score, followed by type C, and finally, type A (**Figure 7B**). In genotyping, genotype C has the highest risk score, followed by genotype B, and finally by genotype A (**Figure 7C**). This is consistent with the previous results of survival analysis based on classification, where the higher the risk score of classification, the worse the prognosis. The waterfall diagram of somatic mutation distribution showed that the top ten genes with the greatest changes in the two risk groups were *VHL, PBRM1, TTN, SETD2, BAP1, MTOR, MUC16, KDM5C, DNAH9*, and *LRP2*. In the high DRG group (**Figure 7E**), the most frequently mutated genes were *VHL* (42%), *PBRM1* (33%), *TTN* (16%), *SETD2* (16%), *BAP1* (12%), and *MTOR* (10%). In the low DRG group (**Figure 7F**), the most frequently mutated genes were *VHL* (41%), *PBRM1* (39%), and *TTN* (15%). At the same time, there was a statistically significant difference in tumor mutation load between the high-risk and low-risk groups, with TBM in the high-risk group being higher than that in the low-risk group (**Figure 7G**). The GSEA enrichment analysis results of high and low-risk groups show that the high-risk groups are mainly enriched in complement activity, phagocytosis recognition, immunoglobulin complex, immunoglobulin complex circulation, and antigen-binding functions (**Figure 7D**), while the low-risk groups are mainly enriched in spliceosomal SNRNP assembly, spliceosomal TRI-SNRNP complex assembly, apical part of cell, spliceosomal SNRNP complex, spliceosomal TRI-SNRNP complex functions (**Figure 7H**). The results of GSVA are visualized using a thermal map (**Figure 7I**). Next, we screened differentially expressed genes from high and low-risk groups for GO and KEGG function enrichment analysis and observed that these genes were enriched in different functions and pathways. The GO results show that it is mainly enriched in extracellular matrix disassembly, regulation of substrate adhesion-dependent cell spreading, positive regulation of epithelial to mesenchymal transition, regulatory T cell differentiation, actin-myosin filament sliding, immunoglobulin complex, circulating, cytoplasmic vesicle lumen, basement membrane, cluster of actin-based cell projections, high-density lipoprotein particle, sulfur compound binding, endopeptidase inhibitor activity, cysteine-type endopeptidase regulator activity involved in apoptotic process, extracellular matrix structural constituent conferring tensile strength, phospholipase A2 activity (consuming 1,2-dipalmitoylphosphatidylcholine) functions (**Figure 7J**). The KEGG results show that it is mainly enriched in Cytokine-cytokine receptor interaction, Complement and coagulation cascades, Viral protein interaction with cytokine and cytokine receptor, IL-17 signaling pathway, TNF signaling pathway, Protein digestion and absorption, Amoebiasis, Mineral absorption, Rheumatoid arthritis, Pertussis, Cholesterol metabolism pathways (**Figure 7K**).

### Drug sensitivity analysis based on DRG score

**Figure 8:**
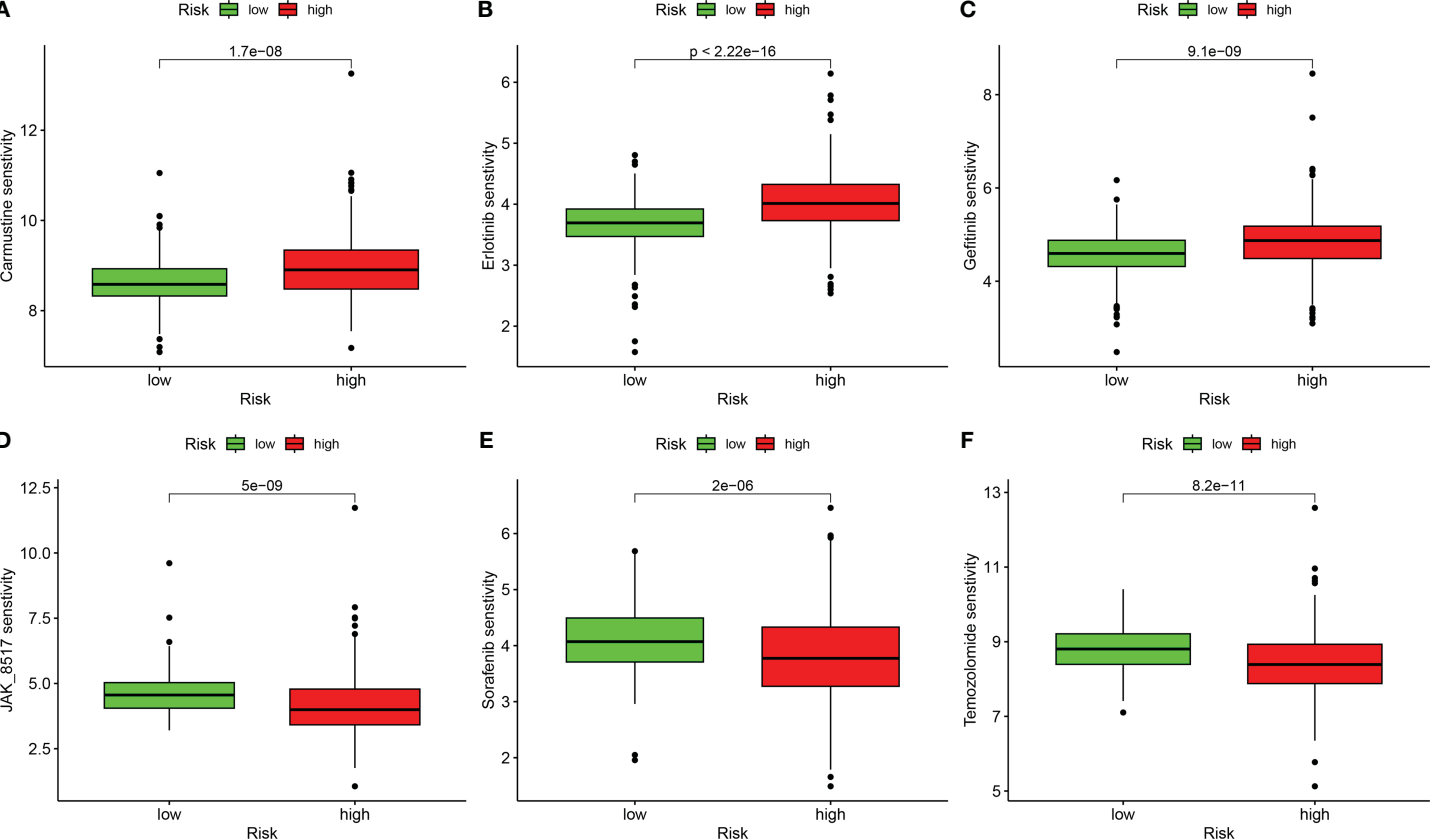
Correlation analysis between risk groups and drug sensitivity. **(A)** Carmustine. **(B)** Erlotinib. **(C)** Gefitinib. **(D)** JAK_8517. **(E)** Sorafenib. **(F)** Temozolomide.

We performed a drug sensitivity analysis for several cancer drugs in the high-risk and low-risk groups, using the IC50 values of the drugs to indicate drug sensitivity, and screened out many drugs with sensitivity differences; we selected only six drugs for demonstration. The high-risk group was more sensitive to Carmustine, Erlotinib, and Gefitinib (**Figures 8A–C**), and the low-risk group was more sensitive to JAK_8517, Sorafenib, and Temozolomide (**Figures 8D–F**). These results suggest that the DRG score is crucial for drug selection.

### *MSH3* could be an important prognostic predictive marker in RCC

**Figure 9:**
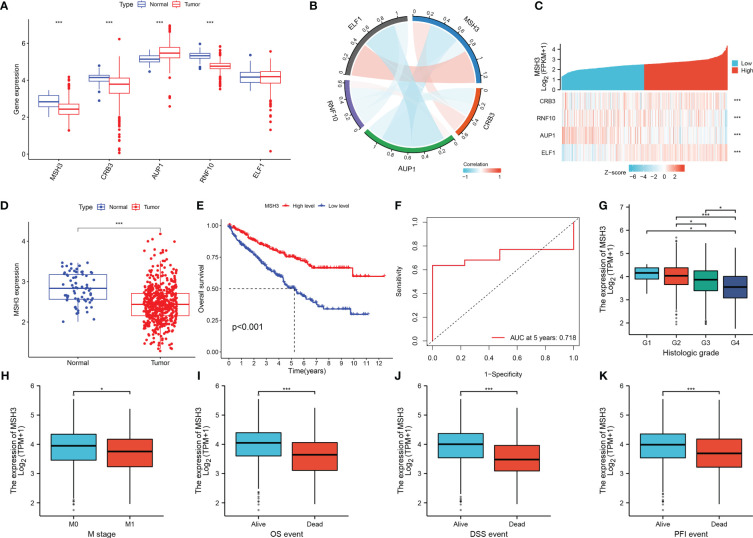
The key gene *MSH3* has an important impact on the prognosis of patients with RCC. **(A)** Expression of five model genes in the TCGA-KIRC dataset. **(B)** Correlation graph between *MSH3* and the other four genes. **(C)** Model gene-related thermograms that vary with *MSH3* expression. **(D)** Expression of *MSH3* in the TCGA-KIRC dataset. **(E)** A survival analysis curve that reflects the relationship between *MSH3* expression levels and OS in patients with RCC. **(F)** ROC curve of *MSH3* predicting 5-year survival probability of RCC patients. **(G–K)** Box diagram showing the correlation between *MSH3* and clinical characteristics of RCC patients. The symbol * indicates p<0.05 and *** indicates p<0.001.

When we investigated the expression of five model genes in RCC, we found that four genes (*MSH3, CRB3, AUP1, RNF10*) had significant expression differences (**Figure 9A**). The correlation chord plot showed a close association between the five model genes (**Figure 9B**). During the study of *MSH3*, it was found that as the expression of *MSH3* increased, the expression of the remaining four genes also changed to varying degrees (**Figure 9C**). *MSH3* expression was downregulated in RCC (**Figure 9D**), and patients with low *MSH3* expression had worse OS (**Figure 9E**), and the ROC curve reveals that the accuracy of using *MSH3* to predict the prognosis of RCC patients is good (**Figure 9F**). In addition, we found that *MSH3* correlated with clinical traits in RCC patients. Patients with higher histological grading had lower *MSH3* expression (**Figure 9G**), patients who developed metastasis had low *MSH3* expression (**Figure 9H**), and patients with low *MSH3* expression had worse OS, DSS, and PFI (**Figures 9I–K**).

**Figure 10:**
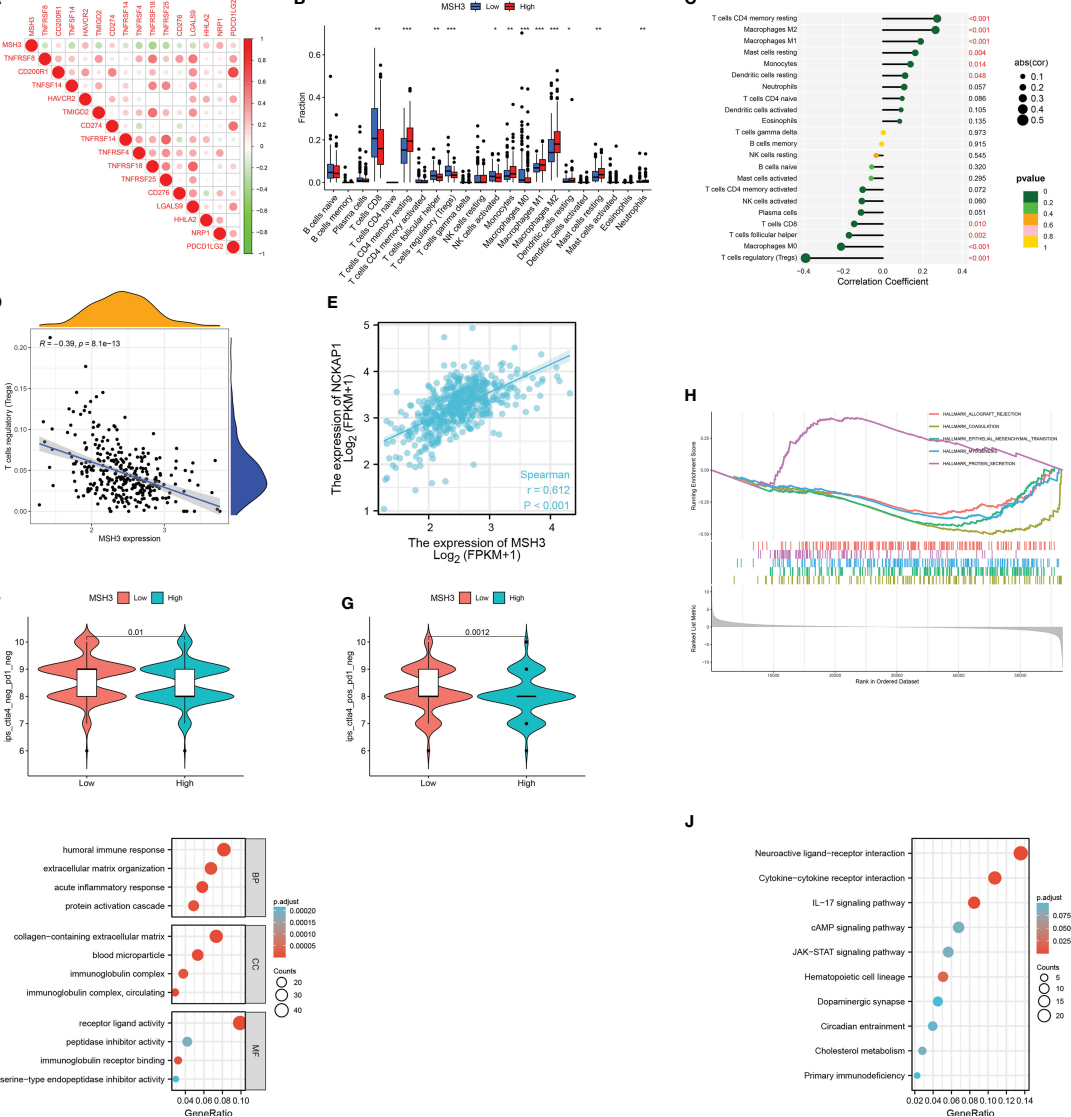
A comprehensive evaluation of bioinformatics related to *MSH3*. **(A)** Immune checkpoint-related genes associated with *MSH3*. The infiltration of immune cells related to *MSH3* is shown in box graph **(B)** and lollipop graph **(C)**. **(D)** Correlation graph between Tregs cells and *MSH3* expression level. **(E)** The result of correlation analysis between the expression of *NCKAP1* and *MSH3*. **(F, G)** Immunotherapy regimens with sensitivity differences at different levels of *MSH3* expression. **(H–J)** *MSH3*-related GSEA enrichment analysis graph **(H)**, *MSH3*-related GO enrichment analysis graph **(I)**, and *MSH3*-related KEGG enrichment analysis graph **(J)**. The symbol * indicates p<0.05, ** indicates p<0.01, and *** indicates p<0.001.

Next, we analyzed the correlation between *MSH3* and immune checkpoint-related genes and found that there are many immune checkpoint gene expression levels associated with *MSH3* (**Figure 10A**). We demonstrated the immune microenvironment infiltration landscape associated with *MSH3* through a box graph and lollipop diagram (**Figures 10B, C**). T cells regulatory (Tregs) are common immune cells in the tumor microenvironment that can suppress immune responses. They promote the occurrence and development of cancer by making the body produce antigen tolerance to tumor cells, thereby allowing tumor cells to escape the immune-killing effect of the body. The correlation analysis between *MSH3* and Tregs showed that the lower the expression of *MSH3*, the higher the Tregs content, indicating a poor prognosis (**Figure 10D**). Moreover, we performed a Spearman correlation analysis of *MSH3* and *NCKAP1*, and the results showed a strong positive correlation between *MSH3* and *NCKAP1* (**Figure 10E**). According to the TCGA database immunotherapy pan-cancer data file, the immunotherapy related to *MSH3* is shown using a violin diagram (**Figures 10F, G**). The GSEA results showed that the group with high expression of *MSH3* was mainly enriched in allograft rejection, while the group with low expression of *MSH3* was mainly enriched in coagulation, epithelial-mesenchymal transition, myogenesis, and protein secretion (**Figure 10H**). We conducted differential analysis on samples with high and low expression levels of *MSH3* and performed functional enrichment analysis on the obtained differential genes. The results of GO analysis showed that these differential genes were mainly enriched in humoral immune response, extracellular matrix organization, acute inflammatory response, protein activation cascade, collagen-containing extracellular matrix, blood microparticle, immunoglobulin complex, immunoglobulin complex, circulating, serine-type endopeptidase inhibitor activity, immunoglobulin receptor binding, peptidase inhibitor activity, receptor-ligand activity functions (**Figure 10I**). KEGG analysis showed that these genes were mainly enriched in Neuroactive ligand-receptor interaction, Cytokine-cytokine receptor interaction, IL-17 signaling pathway, cAMP signaling pathway, JAK-STAT signaling pathway, Hematopoietic cell lineage, Dopaminergic synapse, Circadian entrainment, Cholesterol metabolism, Primary immunodeficiency pathways (**Figure 10J**). In previous studies, *NCKAP1* is a promoter gene of a novel cell death modality called disulfidptosis, and overexpression of *NCKAP1* promotes disulfidptosis in tumor cells, thereby inhibiting tumor cells. Taken together, we believe that *MSH3* can be a key marker for predicting the prognosis of RCC and hope to add to the research on the immune microenvironment, immunotherapy, function, and pathway related to cancer.

### Overexpression of *MSH3* promotes disulfidptosis in 786-O cells and A498 cells under glucose starvation conditions

**Figure 11:**
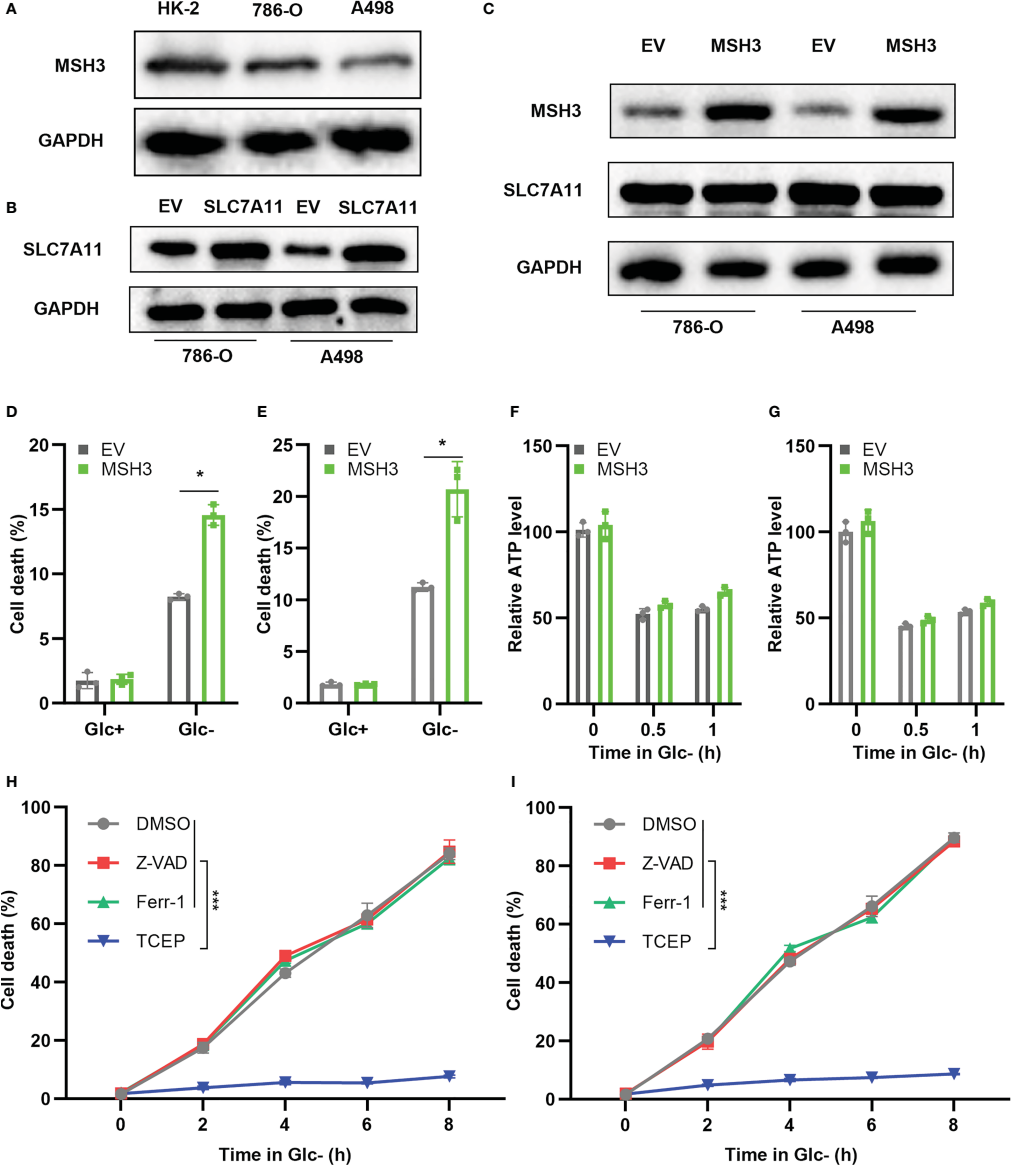
Cell experiments demonstrate that *MSH3* promotes disulfidptosis. **(A)** The results of the western blot showed the expression of *MSH3* in HK-2 and RCC cell lines. **(B)** *SLC7A11* overexpression cell lines were successfully constructed in 786-O cell lines and A498 cell lines, respectively, with the empty vector (EV) as the control group. **(C)** After overexpression of *SLC7A11*, the effect of transfection with *MSH3* on *MSH3* and *SLC7A11* expression. **(D, E)** After overexpression of *SLC7A11*, cell death of the transfected 786-O and A498 cell line in different treatments. **(F, G)** ATP levels of the transfected 786-O and A498cell line under glucose starvation and *SLC7A11* overexpression conditions were measured. **(H, I)** After treatment with different reagents, the cell death of the 786-O and A498 cell line overexpressed with *SLC7A11* and *MSH3* under glucose starvation conditions was measured. The symbol * indicates p<0.05 and *** indicates p<0.001.

To further verify the expression level of *MSH3* in RCC, we examined the protein expression level of MSH3 in human renal cortical proximal tubular epithelial cells and RCC cell lines. The results show that the protein level of *MSH3* in RCC cell lines is much lower (**Figure 11A**), which is consistent with our previous conclusions. We successfully overexpressed *SLC7A11* in 786-O and A498 cell lines (**Figure 11B**). We transfected *MSH3* in the *SLC7A11* overexpressed cells and the result showed that transfection of *MSH3* had no significant effect on the expression of *SLC7A11* (**Figure 11C**). The results of cell death assays showed that overexpression of *MSH3* promoted cell death induced by 786-O Cells (**Figure 11D**) and A498 Cells (**Figure 11E**) under glucose starvation and *SLC7A11* overexpression conditions. Glucose starvation depletes ATP, and glucose starvation does decrease the intracellular relative ATP levels of RCC Cells. However, testing the relative ATP levels of RCC Cells overexpressed with *MSH3* and *SLC7A11* showed that overexpression of *MSH3* had no significant effect on relative ATP levels (**Figures 11F, G**), but significantly promoted cell death under glucose starvation conditions. Therefore, the cell death induced by overexpression of *MSH3* under glucose starvation and *SLC7A11* overexpression conditions is not caused by ATP depletion. To demonstrate whether overexpression of *MSH3* in RCC Cells under glucose starvation and *SLC7A11* overexpression conditions can promote the occurrence of disulfidptosis, and to rule out the occurrence of other known cell death modes. We use a variety of cell death inhibitors, including the ferroptosis inhibitors ferrostatin-1 (Ferr-1) and the apoptosis inhibitor (Z-VAD-FMK). In addition, we use a highly effective disulfide bond reductant tris - (2-carboxyethyl) - phosphine (TCEP). The above reagents were used to treat RCC Cells overexpressed with *MSH3* under glucose starvation and *SLC7A11* overexpression conditions, respectively. The results showed that Ferr-1 and Z-VAD had no salvage effect on the cell death of RCC Cells induced by *MSH3* overexpression under glucose starvation and *SLC7A11* overexpression conditions, while TCEP completely inhibited the cell death of RCC Cells induced by *MSH3* overexpression under glucose starvation and *SLC7A11* overexpression conditions (**Figures 11H, I**). In summary, our results suggest that overexpression of *MSH3* combined with glucose starvation can induce disulfidptosis in *SLC7A11* overexpressed RCC cells.

## Discussion

As one of the most important cancers of the urinary system, RCC has caused indelible suffering and death to patients all over the world, and its poor prognosis has imposed a serious burden on society and the nation. Researchers have been working tirelessly to find biological targets that can predict or improve the prognosis of RCC patients. Encouragingly, more and more articles have been published, such as the cuproptosis-associated 13 genes model as a strong predictor of the efficacy of immunotherapy and targeted therapy for RCC (25), the ferroptosis-related prognostic prediction model for RCC (26), the fatty acid metabolism-related prognostic model of breast cancer (27), and the tumor microenvironment-related prognostic prediction model for non-small cell lung cancer (28). Until now, there are few studies related to disulfidptosis, which is a newly discovered novel cell death modality. Its specific involvement in RCC, the mechanisms of its occurrence and the pathways involved are unknown. With more studies like ours, we believe that the day is approaching when the mechanism of disulfidptosis in RCC will be fully demonstrated and disulfidptosis will be applied to the diagnosis and treatment of RCC to improve the prognosis of RCC patients. Therefore, we investigated the existing 24 DRGs, most of which have differential expression levels compared to normal tissues. By analyzing these genes, we concluded that disulfidptosis could be used as a biomarker for the diagnosis and prognosis of RCC, and a series of studies were carried out.

In our study, we identified three disulfidptosis-related subtypes based on DRGs and then screened the differentially expressed genes among the three subtypes to identify the three genetic subtypes. We found that patients with different subtypes had different OS, immune cell infiltration outcomes and immunotherapy sensitivity. In a step-by-step identification and screening process, we constructed a prognostic model consisting of five genes. And we divided RCC patients into high-risk and low-risk groups according to their risk scores. Then, the utility value of the model in the clinical application of RCC was determined by bioinformatics technology analysis and model validation. The Kaplan-Meier curves showed that the OS of patients in the high-risk group was significantly lower than that of patients in the low-risk group, which highlighted the validity of the model in predicting the prognosis of RCC patients. In the time-dependent ROC curve analysis, the area under the curve of the ROC curve indicated that the prognostic model was highly accurate in predicting RCC prognosis. Consistent results were also obtained in the validation cohort. Interestingly, during the analysis of model genes, we identified the *MSH3* gene, whose low expression was associated with a poor prognosis of RCC. Further studies confirmed that overexpression of *MSH3* under glucose starvation and *SLC7A11* overexpression conditions promoted the development of disulfidptosis in RCC cell lines. The other four model genes (*CRB3, AUP1, RNF10, ELF1*) all have been almost confirmed to function in RCC in different ways. With further studies, our model was superior to traditional clinical features in predicting patient prognosis, indicating good classification of the model. Finally, our analysis confirmed the validity and accuracy of the model in predicting the prognosis of RCC patients.

Notably, we found that *MSH3* may induce disulfidptosis in RCC cell lines by some mechanism under glucose starvation conditions. *MSH3* is a DNA mismatch repair gene that encodes the *MSH3* protein that binds to *MSH2* to form the heterodimer MutSβ, which accomplishes the function of repairing large-scale incorrect base insertion and deletion (29). *MSH3* is associated with the development of various cancers (30), and some studies have shown that *MSH3* is associated with the development of primary nasopharyngeal (31), prostate cancer (32), esophageal cancer (33), colorectal cancer (34), breast cancer (35), while studies on renal cell carcinoma are rare. In our study, we found that *MSH3* expression was reduced in RCC patients and correlated with clinical traits, and patients with lower *MSH3* expression had a worse prognosis. Also, *MSH3* was closely associated with tumor microenvironment infiltration, immune checkpoint genes, and immunotherapy sensitivity. Our study showed that *MSH3* expression was negatively correlated with the amount of Tregs cells, which play a role in promoting tumor development and immune evasion in kidney cancer. Finally, the results of cellular experiments showed that *MSH3* expression was indeed downregulated in RCC cell lines, which is also consistent with our previous findings. After overexpression of *SLC7A11*, the onset of cell death was detected by overexpression of *MSH3* in glucose-starved treated RCC Cells and was rescued after treatment with TCEP. Therefore, we suggest that *MSH3* may be a potential prognostic biomarker for RCC patients. However, the biological role of *MSH3* in RCC has not been elucidated, and the mechanism of how disulfidptosis is induced also needs further experimental investigation.

It is well known that tumor cells together with lymphocytes, immune cells and tumor cell blood vessels surrounding tumor cells build the tumor microenvironment (36–38).. A considerable number of studies have confirmed that tumor microenvironment infiltration has a dramatic impact on tumor formation and progression as well as treatment resistance (39–41). Our study demonstrates the immune landscape of tumor microenvironment infiltration under different subtypes and different risk groupings, and their relevance to clinical traits and immunotherapy. Based on the results that tumor microenvironmental infiltration and immunotherapy differ significantly across subtypes and different risk subgroups, we suggest that the DRG risk score model is crucial for the development, progression, and treatment of RCC. Among the disulfidptosis subtypes, subtype B had a higher level of immune cell infiltration than the other subtypes and was associated with the worst prognosis. Similarly, the proportion of some cancer-promoting tumor-infiltrating immune cells such as Tregs cells increases with increasing risk scores (42). It had been suggested that TIDE scores tend to reveal the likelihood of tumor immune escape, so a higher TIDE score in the high-risk group may explain the worse prognosis for patients in this group (43). As we have observed, some studies have shown the potential value of disulfides in tumor diagnosis and treatment. For example, thiol/disulfide homeostasis (TDH) alteration could be used as an early diagnostic and therapeutic target for ccRCC (44). In addition, a link between disulfidptosis and tumor immunity has been found (45). Our results are consistent with previous studies, but there are still too few studies on the relationship between disulfidptosis and tumor immunity, and the mechanism of this needs to be further explored.

## Conclusion

In this study, we investigated the effect of DRGs on RCC prognosis, identified disulfidptosis-related subtypes, and demonstrated the immune landscape of different subtypes. We then developed a new prognostic prediction model based on five DRGs and confirmed that it can accurately predict OS in RCC patients, demonstrating differences in clinical features, somatic cell variants, tumor microenvironment infiltration, and drug sensitivity associated with risk scores. Finally, we also identified the *MSH3* gene, which is associated with prognosis and disulfidptosis in RCC patients. In summary, Our study may provide new insights into the subtype and treatment of RCC.

## Data availability statement

The datasets presented in this study can be found in online repositories. The names of the repository/repositories and accession number(s) can be found within the article/**Supplementary Material**.

## Author contributions

KX, YZ, and ZY contributed to the conception and design of this study. YW, YL, and QQ collected and analyzed the data. KX, YZ, and YW drafted the original manuscript. YD, ZC, XL embellished and revised the manuscript. This manuscript has been read and approved by all authors. All authors contributed to the article and approved the submitted version.
